# Unmanned aerial vehicles in prehospital emergency care: current evidence, technological advances, and future perspectives

**DOI:** 10.3389/fpubh.2025.1739160

**Published:** 2026-01-13

**Authors:** Yanguoer Zhang, Junjie Cao, Sheng Ye

**Affiliations:** 1 The Second Affiliated Hospital of Wannan Medical College, Wuhu, China; 2 Wannan Medical College, Wuhu, China

**Keywords:** disaster medicine, out-of-hospital cardiac arrest, prehospital emergency care, trauma care, unmanned aerial vehicles

## Abstract

**Background:**

Timely prehospital intervention is essential for improving patient outcomes, yet conventional ambulance-based systems often face delays in remote, congested, or disaster-affected settings. Unmanned aerial vehicles (UAVs) offer a novel, traffic-independent solution with high operational flexibility and rapid deployment capacity. This review aims to summarize current evidence, technological progress, and future challenges of UAVs applications in prehospital emergency care.

**Methods:**

A systematic literature search was performed in PubMed, ISI Web of Science, and EBSCO Essentials for studies published up to September 2025. Relevant clinical trials, pilot projects, and policy documents were also reviewed to capture the latest developments in UAV-based prehospital interventions.

**Results:**

53 studies met the inclusion criteria and were included in the final analysis. UAVs have been implemented across several domains, including out-of-hospital cardiac arrest, trauma care, emergency medical logistics, and disaster response. Key applications involve rapid automated external defibrillator (AED) delivery, transport of blood products and critical drugs, and aerial imaging for scene assessment and command coordination. Despite growing feasibility evidence, broad implementation is hindered by technical reliability, regulatory restrictions, and limited funding frameworks.

**Conclusion:**

UAVs demonstrate significant potential to enhance the speed, coverage, and coordination of prehospital emergency care. Future research should focus on integrating UAVs within established emergency medical service networks, developing unified policy and airspace regulations, and validating cost-effectiveness and clinical impact through large-scale, real-world studies.

## 1 Introduction

Prehospital emergency care (PHEC), as an integral component of the emergency medical system, constitutes the critical initial link bridging patients and definitive in-hospital treatment. The operational efficiency and quality of PHEC interventions are decisive determinants that directly influence both short-term survival and long-term functional outcomes in critical patients. PHEC encompasses a broad spectrum of emergencies–from out-of-hospital cardiac arrest (OHCA), typically triggered by cardiovascular or cerebrovascular events and necessitating prompt cardiopulmonary resuscitation and defibrillation (1), to severe trauma resulting from road traffic collisions (2, 3), falls from height, or other high-energy impacts that cause acute organ injury or massive hemorrhage (4). It further extends to large-scale disaster scenarios, where mass-casualty incidents caused by earthquakes, floods, fires, or other catastrophes generate complex injury patterns and place overwhelming demands on emergency resource (5). By responding to these diverse and time-critical emergencies, PHEC serves as the frontline safeguard of the acute care system, underscoring the urgent need for innovative strategies and emerging technologies–such as unmanned aerial vehicles (UAVs)–to enhance its efficiency, reach, and overall effectiveness.

In recent years, UAV technology has evolved rapidly, from experimental prototypes to large-scale industrial deployment, emerging as a pivotal driver of technological innovation. This advancement is fueled by the inherent advantages of UAVs, which include operational agility, cost-effectiveness, and flexible deployment capabilities. These characteristics collectively make UAVs well-suited for diverse practical applications. Early implementations have already demonstrated considerable potential across multiple sectors, such as precision agriculture (6, 7), industrial operations (8), and public safety (9, 10). Furthermore, the parallel maturation of fifth-generation (5G) communication networks has significantly expanded the operational boundaries of UAVs. Owing to the high bandwidth and ultra-low latency of 5G, UAVs are now capable of transmitting high-definition images and video streams in real time, thereby enabling remote terminals to access sensor-acquired field data with unprecedented clarity (11). In addition, the stability and reliability of 5G signals improve remote control precision and operational efficiency, while simultaneously reducing task failures caused by signal interruptions. Consequently, these technological advances have collectively transformed UAVs from isolated operational tools into intelligent, interactive platforms, establishing a robust foundation for their integration into complex and mission-critical scenarios, including emergency response and prehospital medical support (12).

## 2 Methodology

The systematic literature search was conducted to review the current research and applications of UAVs in prehospital medicine. The search covered five major electronic databases: PubMed (MEDLINE), ISI Web of Science (WOS), EBSCO Essentials (EBSCO), WANFANG Data, and VIP Database. The search timeframe is from the inception of each database to September 30, 2025, with language restrictions limited to Chinese and English literature. Relevant keywords were used, including “out-of-hospital cardiac arrest AND UAV,” “trauma rescue AND UAV,” “critical care AND UAV,” “internal medicine emergencies AND UAV,” “disaster medicine AND UAV,” “AED AND UAV,” “blood product transport AND UAV,” “diagnostic forward deployment AND UAV,” “mobile communication base station AND UAV,” among others.

Literature selection was conducted in a stepwise and systematic manner. Initially, titles and abstracts were screened to identify studies with potential relevance, after which full-text articles were retrieved and reviewed in detail to confirm eligibility. Throughout this process, literature screening strictly adhered to predefined inclusion and exclusion criteria. Specifically, randomized controlled trials (RCTs), cohort studies, controlled studies, simulation modeling studies, technical feasibility reports, and prospective pilot studies were considered eligible for inclusion. In contrast, non–peer-reviewed preprints, editorials, commentaries, news briefs, conference abstracts, and case reports lacking clear methodological descriptions were systematically excluded.

**Figure 1:**
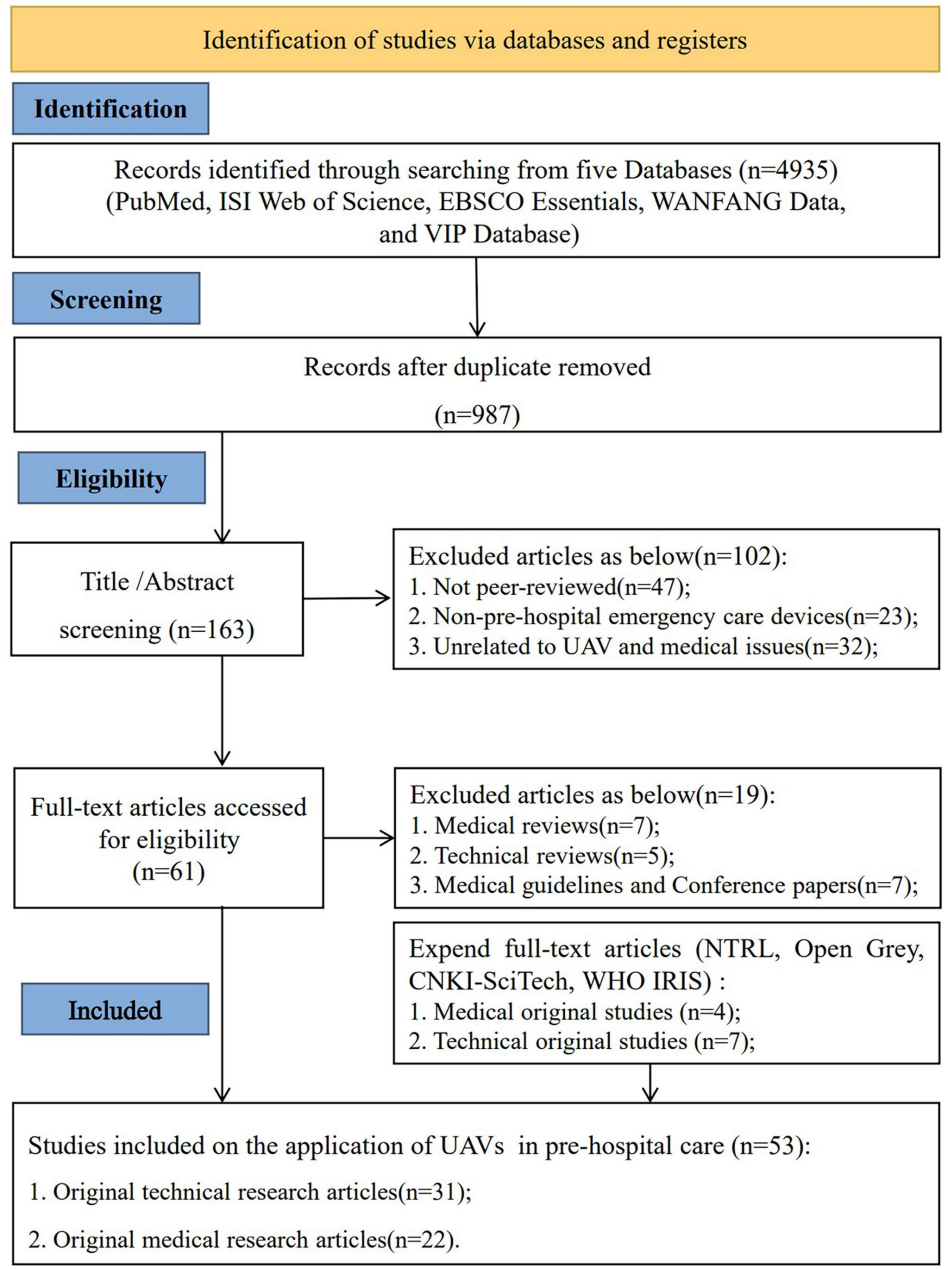
PRISMA flow diagram illustrating the study selection and inclusion process for the systematic review.

The initial database search yielded 4,935 records. After removal of duplicates, 163 articles were screened for relevance, of which 61 full-text articles met the preliminary inclusion criteria and were assessed in detail. During full-text evaluation, studies classified as medical reviews (*n* = 7), technical reviews (*n* = 5), and medical guidelines or conference papers (*n* = 7) were excluded, as they did not meet the criteria for original research. To ensure comprehensive coverage, an expanded full-text search was subsequently conducted using additional sources, including the National Technical Reports Library (NTRL), CNKI-SciTech, and the WHO Institutional Repository for Information Sharing (WHO IRIS). Through this process, 11 additional eligible original research articles were identified and included. Consequently, a total of 53 original studies were included in the final analysis, comprising 31 original technical research articles and 22 original medical research articles (Figure 1).

## 3 Applications of UAVs in prehospital medicine

### 3.1 UAVs for OHCA and AED delivery

OHCA remains a major contributor to global morbidity and mortality, with an incidence of 142.6 per 100,000 population in the United States (13) and approximately 121.5 per 100,000 in Mainland China (14). Given this substantial burden, the timely delivery of life-saving interventions is essential, as survival outcomes are highly time-dependent. Evidence demonstrates that defibrillation within 60 s of collapse can yield survival rates approaching 90% (15), whereas intervention within three minutes is associated with approximately 70% survival. However, with each additional minute of delay, the likelihood of survival declines by 7–10% (16). Against this backdrop, the development of innovative strategies to shorten time-to-defibrillation has become a central priority in prehospital emergency medicine.

In response to these challenges, UAV-delivered AED systems have emerged as a promising solution to overcome the inherent limitations of conventional ambulance-based response. Early real-world studies conducted in Sweden and Denmark demonstrated both the feasibility and operational safety of UAV-delivered AED. For instance, Schierbeck and colleagues reported a 92% operational success rate in a region serving 80,000 residents (17), while Jakobsen and co-workers confirmed the effectiveness of UAV-delivered AED within a 6 km urban airspace covering 110,000 residents (18). Importantly, comparative investigations further emphasized the value of strategic UAV placement. In a Swedish study deploying five UAVs across segregated airspaces, AEDs arrived ahead of ambulances in 67% of dual-response cases, yielding a median time advantage of 3 min and 14 s (19). These temporal gains translated directly into improved clinical outcomes, as AED delivery within five minutes was associated with a 3.3-fold increase in neurologically intact survival compared with longer delays (20).

Building on this evidence, recent research has increasingly shifted from feasibility testing to optimization of UAV deployment strategies. Spatial analyses conducted in Stockholm revealed distinct patterns across urban and rural settings. Centralized UAV placement in urban areas, where OHCA incidence is high and ambulance delays are relatively short, reduced median response times by 1.5 min. Conversely, more peripheral deployment in rural areas, characterized by lower incidence but longer delays, achieved reductions of up to 19 min (21). Complementing these geographic approaches, Boutilier and colleagues developed mathematical optimization models that integrate facility location theory and queuing principles. Their findings suggest that moderately scaled UAV networks can shorten response times by up to 76%, corresponding to an estimated 144 additional lives saved annually (22). Collectively, these results highlight that UAV-based AED systems are not only technically feasible but also operationally transformative when adapted to local epidemiology and emergency medical service (EMS) infrastructure.

Nevertheless, the practical effectiveness of UAV-AED networks ultimately depends on reliable delivery modalities. Once reaching an incident site, UAVs must decide between precision landings and alternative methods such as parachute deployment, tethered release, or winch-assisted delivery (23). Advances in vision-based navigation, including fiducial marker recognition and ArUco-based pose estimation, have enabled centimeter-level landing accuracy, which is particularly critical in complex urban or obstructed environments (24). Primary modalities for AED delivery via UAVs include parachute deployment from altitudes of 25 meters or higher, remote-release systems that drop payloads from heights of 3–4 meters, and direct landings on level terrain where AEDs are mounted on top of the UAV and access is permitted only after rotor stoppage. Direct landing has often been regarded as providing the best balance between safety and operational efficiency. In addition, empirical studies by Anand A. et al. (25) have evaluated winch-assisted delivery, showing that it achieves superior precision, with accuracies of 95 and 92% respectively, though at the cost of extended deployment times of approximately 120 and 110 s. By contrast, free-fall drops allow for rapid AED delivery within 50 to 60 s, but precision is compromised, with accuracies of 85 and 80%. Tethered release systems demonstrate intermediate performance, with accuracy of about 88% and average delivery times of 90 s (25, 26). These comparative findings indicate that modality selection should be tailored to the specific emergency scenario, weighing the trade-off between speed and precision while also accounting for terrain constraints. Winch delivery appears most suitable for environments requiring high accuracy, free-fall drops for situations demanding the fastest possible response, and tethered release as a balanced option in contexts where both timeliness and precision are critical.

Despite notable progress, important limitations remain. A significant proportion of studies has excluded nocturnal operations, even though low-light conditions, adverse weather, and regulatory restrictions may substantially affect UAV flight safety and delivery accuracy. Encouragingly, pilot studies in Germany have shown that nighttime missions can achieve similar overall response times to daytime operations, with even slightly faster landings despite reduced visibility (27). However, these experiments were performed under controlled meteorological conditions and within specific regulatory exemptions, indicating that further validation is necessary before widespread adoption. Taken together, the growing body of evidence underscores the transformative potential of UAV-based AED delivery systems for OHCA management, while also highlighting the operational, environmental, and regulatory challenges that must be systematically addressed to enable scalable real-world implementation.

### 3.2 UAVs in trauma and hemorrhage control

Trauma remains a leading global health challenge, causing approximately 4.4 million deaths annually (28). Trauma-induced coagulopathy (TIC) occurs in 25–35% of severely injured patients, significantly driving early mortality and ranking as a leading cause of preventable death (29). UAVs offer expansive and multifaceted applications to enhance rescue efficiency and improve clinical outcomes. Leveraging their rapid deployment and spatial maneuverability, UAVs can deliver critical medical supplies–including AEDs, hemostatic dressings, and emergency drugs–directly to incident sites. Mesar et al. (30) demonstrated UAVs could transport 4.5 kg payloads (tourniquets, dressings, analgesics, blood products) within 20.77 min to 1-meter precision, significantly outperforming ground transport. In maritime scenarios, Seguin et al. (31) showed UAVs delivered flotation devices in 81 ± 39 s under moderate sea conditions and 99 ± 34 s in adverse conditions, compared to 179 ± 78 and 198 ± 130 s by lifeguards, respectively (*p* < 0.001), while performance remained unaffected by weather. UAVs also transform real-time monitoring in trauma care through the integration of advanced sensing technologies. Huang et al. (32) integrated 4D millimeter-wave radar into UAVs and proposed an adaptive 3D human point cloud generation method (SA-m Point). This approach effectively mitigates issues such as phantom targets and misclassification caused by multipath effects by leveraging micro-motion and respiration characteristics. Experimental results showed that the method achieved 97.94%-point cloud accuracy, increased point yield by 87.94%, and reduced runtime by 11.41% compared to existing algorithms. It enables precise target alignment and continuous cardiopulmonary signal acquisition during UAV hovering, effectively eliminating motion artifacts. In addition to physiological assessment, UAVs also play a critical role in post-trauma emergency coordination. De Oliveira et al. (33) developed a deep learning framework employing low-cost RGB and thermal imaging cameras mounted on UAVs to detect pedestrians in aerial imagery, thereby facilitating real-time traffic management and rescue route optimization. Complementing this, Hoshiba et al. (34) designed an embedded microphone array system that enables UAVs to localize acoustic sources in outdoor environments, a function that supports traffic guidance and on-site command in complex operational scenarios. Collectively, these studies not only demonstrate innovative UAV designs but also provide robust empirical evidence, thereby laying a strong foundation for and helping to advance future research in this domain.

A particularly critical challenge is hemorrhage control and blood logistics, since uncontrolled bleeding is a leading cause of early trauma mortality (28). Amukele et al. (35) demonstrated UAV- transported whole blood, plasma, and platelets preserved key biochemical parameters (erythrocyte hemolysis, platelet aggregation, coagulation factor activity) with no statistical differences compared to ground transport (*p* > 0.05). To optimize transport under diverse environments, Wen T. established tables specifying ideal ratios of blood weight to heating/cooling agents, ensuring storage within 2–10 °C despite ambient variations from −5 °C to >15 °C (36). Furthermore, Homier et al. (37) validated UAVs with 6.4 kg payloads completing 9 flights at 10 m/s, with mean duration 17.06 ± 0.04 min–over 11 min faster than ground transport (28.54 ± 1.12 min, *p* < 0.0001) while maintaining thermal stability: packed RBCs deviated ≤5% from 4.4 °C, platelets showed only ±0.3% variation at 21.6 °C, and frozen plasma remained at −17 °C to −16 °C. However, scalability challenges persist, as Xia et al. (38) highlighted capacity constraints under multi-patient demand despite hybrid UAV–truck deployment, underscoring the need for further optimization.

The existing evidence clearly indicates that the application of UAVs in trauma emergency care is evolving along three interconnected trajectories: from enhancing the speed of critical supply delivery to meet extreme timeliness requirements, to integrating monitoring and sensing functions to support early on-scene assessment and decision-making, and further to validating the reliability of transporting special medical supplies such as blood to extend the life-support chain. These advancements collectively point to a core conclusion: UAVs are becoming a spatially mobile node capable of effectively compressing prehospital time and enhancing on-site treatment capacity. However, their current role primarily manifests as a capacity supplement to the existing emergency response systems. The ultimate realization of large-scale application remains contingent upon breakthroughs in common bottlenecks, such as payload endurance, system resilience, and standardized integration.

### 3.3 UAVs in critical illness management

Critical care delivery continues to face formidable challenges, particularly during prehospital response and early transport phases. Geographic and infrastructural barriers, the strict time sensitivity of the “golden hour” delays in delivering life-saving resources, and gaps in situational awareness within high-risk environments, combined with the persistent risks faced by first responders, create bottlenecks that conventional rescue approaches struggle to overcome (39, 40). Against this backdrop, the integration of UAV technology has emerged as an innovative solution to address these critical limitations. Sheng et al. (41) developed a system that enabled automated, targeted transdermal delivery of emergency drugs using UAVs equipped with contact-triggered microneedle applicators under the uFAST framework, which demonstrated effective dual-phase release and a favorable safety profile of glucagon microneedle patches in a porcine hypoglycaemia model, establishing UAVs as a universal emergency response tool in acute disease management. Similarly, UAV–enabled antivenom delivery in rural Odisha, India, reduced median access time from 4.2 h to just 18 min, resulting in a decline in snakebite mortality from 28.7 to 6.9% (42). Cox (43) demonstrated through a network model of 2,327 opioid overdose incidents that increasing UAV base station density significantly reduced response times, and shortened the median response interval by 4 min and 38 s compared to ambulance dispatch, extending county coverage to 64.2%. These findings collectively suggest that UAV-based pharmaceutical delivery is evolving from pilot testing to scalable implementation and is likely to become an integral component of future healthcare infrastructure.

In addition to pharmaceuticals, UAVs are increasingly used in emergency equipment deployment. Fakhrulddin et al. (44) designed a cardiac monitoring platform for geriatric patients with cardiovascular disease that incorporated UAV-delivered emergency kits. Compared with ambulances, UAV delivery achieved a mean time saving of 105 s and reduced overall response times by 31.81%, primarily due to the accelerated transport of supplies directly to patients. Likewise, Bäckman et al. (45) demonstrated the advantages of UAV deployment in drowning scenarios. Across 30 simulated rescues, UAVs delivered flotation devices within a median of 30 s (IQR 24–32), which was significantly shorter than the 65 s (IQR 60–77) required for traditional swimming rescues (*p* < 0.001). More importantly, the inflatable rings reached within one meter of victims in 95% of trials, confirming UAVs as a highly accurate and efficient flotation delivery method. Extending beyond immediate rescue, UAVs also show promise in critical transplantation logistics. Scalea et al. (46) reported the first successful human kidney transport by UAV over a distance of 4.8 km in 10 min, with histopathological and molecular analyses confirming complete organ viability, thereby establishing a new technological pathway to reduce cold ischemic time.

Beyond material delivery, UAVs also advance prehospital diagnostics through high-definition imaging, multispectral analysis, and real-time data transmission. Claesson et al. (21) validated this paradigm by demonstrating UAV-enabled real-time ECG transmission from the field to hospitals, enabling early cardiac assessment prior to patient arrival. This accelerated response allows medical teams to begin preliminary diagnoses before patient arrival, significantly improving clinical decision-making. Al-Naji et al. proposed a technique that used hovering UAV video imaging and CEEMDAN-CCA filtering to measure heart rate (error: 0.6 bpm) and respiratory rate (error: 0.34 bpm) from 3 meters within 1 min, achieving accuracy comparable to in-hospital contact monitors. It requires no electrodes, preventing secondary injury in sensitive cases like burns or infants. The algorithm is robust to motion, light changes, and body sway, making it suitable for complex scenes (e.g., night, ruins). Real-time data transmission via onboard Wi-Fi enables emergency centers to conduct remote triage, initiate interventions, and pre-activate resources like catheterization labs or ECPR teams before patient arrival, offering a viable path for pre-hospital diagnostic advancement (47). Furthermore, Roberts et al. (48) reported that UAVs consistently arrived at incident sites within 3 to 5 min, which was 10 to 20 min faster than ambulances. The integration of real-time vital sign transmission further enabled prehospital assessment, collectively enhancing response efficacy and improving survival rates by 18 to 35%.

Above all, these empirical findings demonstrate that UAV integration not only overcomes the spatiotemporal limitations of traditional rescue systems but also shifts the recognition and intervention nodes of critical care forward in time. This forward-shift fundamentally redefines prehospital critical care, positioning UAVs as a transformative tool in the diagnosis, treatment, and survival of critically ill and injured patients. Its core value is primarily reflected in the following aspects: significantly improving emergency response efficiency, reducing the inherent risks to rescue personnel, optimizing the spatial allocation of limited medical resources, and demonstrating the potential to improve clinical outcomes in specific emergencies such as poisoning and cardiac arrest. Looking ahead, technological evolution will focus more on the integration of multimodal sensing, autonomous intelligent decision-making, and its deep integration with existing medical systems. However, realizing its full potential still depends on the continuous deepening of interdisciplinary research, the validation of cost-effectiveness across different contexts, and the simultaneous construction of regulatory and ethical frameworks adaptable to diverse developmental environments. Only through such systematic advancement can this technology ultimately be promoted to integrate equitably and accessibly into the global emergency response system.

### 3.4 UAVs in disaster triage and communication

When mass casualty incidents (MCIs) and other large-scale emergencies occur, systematic triage is essential, as it can reduce overall mortality by 20–30% and is recognized by the World Health Organization (WHO) as a core component of disaster medical response (49). In recent years, rapid advances in UAV technology have prompted increasing research into UAV-assisted triage at disaster sites, demonstrating both feasibility and potential clinical value. Lu et al. (50) developed a novel triage system that integrates UAVs with artificial intelligence, enabling fusion of multimodal aerial data to dynamically prioritize victims. In practice, once an incident occurs, the emergency command center can dispatch the nearest pre-deployed medical UAVs, which then utilize embedded triage algorithm modules such as Simple Triage and Rapid Treatment (START) to provide automated voice-guided support for on-site responders. Building on this, Jain T. et al. conducted a randomized study involving 40 paramedic students from the Dutch College Paramedicine Program in Canada (51). In a simulated motor vehicle collision scenario, participants were randomly assigned to UAV-assisted triage or standard protocol groups during both daytime and nighttime trials. The results demonstrated that the UAV-assisted group achieved significantly shorter triage completion times, averaging 3.63 min (95% CI: 2.45–4.85 min, *p* = 0.002) during the day and 3.49 min (95% CI: 2.08–6.06 min, *p* = 0.002) at night, compared with the control group. However, there were no observed differences in patient evacuation time, on-scene duration, or triage sequencing, and both groups maintained 100% accuracy. These findings confirm the feasibility of UAV-assisted triage, although the time savings, while statistically significant, did not translate into clinically meaningful improvements in evacuation workflows. Nonetheless, UAVs offer the ability to remotely classify patients, bypass ground traffic congestion, and accelerate response times, although these advantages also place higher technical demands on personnel tasked with operating the systems.

Beyond triage, UAVs play a crucial role in sustaining communication during disasters. In large-scale natural events such as earthquakes or tsunamis, traditional communication infrastructure is often severely compromised. Choi et al. (52) developed UAV-mounted mobile base stations equipped with Public Safety LTE technology, which were shown in outdoor validation trials to function effectively as independent communication nodes, achieving downlink speeds of 22 Mbps and uplink speeds of 8 Mbps within a one-kilometer range. Similarly, McRae et al. (53) evaluated aerial UAV relay systems in southern Utah to restore radio communication between incident command and field teams. In ten previously identified communication dead zones, UAV relays flown at an altitude of 122 meters successfully restored communication in all cases, with an average restoration time of 6.5 ± 1.1 min (range: 4.4–9.3 min). These studies demonstrate that UAV-based communication platforms can significantly enhance disaster monitoring, optimize emergency coordination, and reduce operational risks.

In addition to triage and communication, UAVs are increasingly used in search and rescue operations in complex and high-risk terrains. Traditional manual approaches often expose responders to secondary hazards and limit search efficiency, whereas UAVs equipped with advanced sensing systems offer safer and more effective alternatives. Lygouras E. and colleagues developed a UAV with embedded vision systems and deep learning algorithms capable of autonomous personnel detection during search operations (54). Their system achieved 90.2% detection accuracy in complex environments, with a processing latency of 0.07 s per frame and coverage of two square kilometres in ten minutes, and the system identified 14 of 15 mock casualties (6.7% miss rate) in simulated disaster scenarios. Complementing these advances, Al-Kaff A. and colleagues developed an appearance-based tracking algorithm that allowed UAVs to transmit high-resolution video for real-time victim localization (55). Their system achieved a detection accuracy of 82.1%, covered three square kilometers within 15 min, and successfully identified 17 of 20 simulated casualties. Hoshiba K. further expanded UAV capabilities by integrating embedded microphone arrays for acoustic localization, achieving 87.6% detection accuracy and successfully identifying 11 of 12 mock casualties under challenging outdoor conditions (34). In summary, these studies confirm that UAV deployment in disaster-area search and rescue fundamentally transforms traditional paradigms by reducing responder exposure, improving operational safety, and enhancing coverage and accuracy. Looking ahead, continued advancements in UAV endurance, adaptability to adverse environments, and multi-UAV coordination are expected to further expand their utility, ultimately advancing disaster response systems toward more intelligent, AI-driven, and precision-based rescue models.

## 4 Challenges and limitations

UAVs demonstrate substantial potential in prehospital emergency care; however, their extensive deployment and sustained advancement still confront multifaceted challenges.

### 4.1 Technical level

#### 4.1.1 Payload capacity

Payload capacity represents a fundamental constraint on the range and volume of medical supplies that UAVs can deliver in prehospital emergencies. The requirements in these situations often extend beyond lightweight items such as pharmaceuticals and hemostatic kits to include heavier equipment such as cardiac defibrillators and portable ventilators. These demands exceed the 6.4 kilogram payload ceiling of most contemporary systems and create critical operational gaps in complex resuscitation scenarios (56). In cases involving polytrauma patients, where defibrillators, oxygen cylinders, and other equipment must be transported simultaneously, conventional UAVs may fail to take off due to payload overload or may experience a sharp decline in endurance caused by excessive weight. To address these challenges, researchers explored high-strength lightweight composite materials for increased load-bearing capacity with reduced weight. At the same time, modifications to propulsion systems were proposed to improve efficiency and thereby expand the maximum effective payload capacity (57, 58).

#### 4.1.2 Endurance

The service coverage radius of UAVs is primarily constrained by their maximum operational range. Current systems remain limited by battery energy density and propulsion efficiency, which makes it difficult to serve remote regions or conduct cross-regional emergency missions effectively (59). Overcoming these challenges requires advancements that extend beyond incremental improvements in battery technology and must also include the development of optimized route planning strategies to reduce unnecessary energy expenditure and extend mission range. Zhang et al. (60) have explored a “UAV relay mode” in which temporary refuelling or recharging stations are established at critical nodes, enabling coordinated multi-UAV handovers that extend the service radius. In addition, optimizing route planning algorithms minimizes redundant flight trajectories and improves per-kilometer energy utilization, thereby enhancing overall endurance and mission efficiency (61).

#### 4.1.3 Landing and delivery modalities

Landing efficiency and safety play a critical role in ensuring smooth emergency procedural transitions. In densely populated urban areas where dedicated vertiports are absent, spatial restrictions create major barriers to UAV landing (62). Similarly, in complex wilderness terrains such as mountainous or aquatic environments, irregular ground surfaces may compromise airframe stability or cause payload destabilization during landing operations (63). In response to these challenges, Palafox et al. (64) developed an adaptive landing system that integrated a vision–inertial fusion framework, allowing UAVs to identify suitable landing zones in real time and achieve secure touchdown on dynamic platforms and uneven terrains. When viable landing zones are entirely absent, UAVs can instead deliver supplies through tethered release mechanisms followed by autonomous ascent, which reduces ground contact risks while maintaining operational continuity.

Beyond landing, delivery stability is equally important for preserving the integrity of medical payloads. Conventional aerial delivery methods such as parachute deployment often suffer from significant trajectory deviation in high-wind conditions exceeding 8 m/s. At the same time, traditional drop mechanisms carry the risk of damaging fragile medical items, including glass vials and precision monitoring devices (65). To mitigate these risks, researchers explored the use of specialized buffer systems incorporating materials such as paper honeycomb, aluminium honeycomb and expanded polystyrene, which combined with pneumatic damping and hydraulic buffering technologies, provided effective impact attenuation and helped ensure the safe preservation of medical supplies during aerial delivery (66).

#### 4.1.4 Weather factors

Adverse meteorological conditions represent one of the major operational challenges for UAV deployment. High-velocity winds can destabilize the aircraft’s attitude, heavy rainfall may compromise electronic components, dense fog often obstructs visual sensors and undermines positioning accuracy, and extreme temperatures can significantly reduce battery capacity and avionics reliability (67). For instance, during pre-typhoon gales, UAVs are prone to drifting off course or even suffering catastrophic crashes. In addition, subzero conditions sharply reduce battery endurance, making it difficult to complete missions. To address these risks, enhanced fuselage sealing and waterproofing improved resistance against precipitation and corrosion, while the integration of meteorological sensors enables autonomous return-to-base decisions or obstacle-avoidance maneuvers in response to real-time wind and visibility data (68). Furthermore, the development of specialized UAV variants optimized for extreme-weather operations offers a more robust approach to sustaining functionality in adverse environments.

#### 4.1.5 Collision avoidance system

The reliability of collision avoidance systems also presents a critical challenge for UAV deployment in complex environments. Current technologies are often validated only in laboratory or simplified test settings, which fail to capture the unpredictability of real-world operations. As a result, existing systems show limited accuracy and delayed responsiveness when confronted with sudden obstacles such as intruding birds, unmarked power lines, or fast-moving vehicles (69). For instance, in urban low-altitude flights, an unexpected bicycle crossing the flight path could result in collisions due to insufficient recognition speed. Addressing these shortcomings requires a combination of expanded real-world testing and advanced simulation approaches. Broader testing in diverse contexts–including high-traffic corridors, avian habitats, and densely constructed zones–could provide critical collision data under multifactorial interference, supporting algorithmic refinement for real-time responsiveness. At the same time, the integration of digital twinning technology offers a complementary pathway, bridging gaps left by empirical trials and improving the overall robustness of sense-and-avoid systems (70).

#### 4.1.6 Maintenance of drugs and instrument

The stability of medical supplies is essential for effective emergency intervention, as certain pharmaceuticals, including insulin and vaccines, require strict low-temperature preservation. However, UAV cargo compartments are often exposed to environmental fluctuations during flight, which could compromise temperature-sensitive materials. Pamula and Ramachandran (71) emphasize that whole-blood products and refrigerated vaccines must be kept within 2–8 °C; any deviation risks hemolysis or irreversible loss of potency. To address these challenges, UAVs could be fitted with thermally sealed cargo compartments that incorporate semiconductor-based thermoelectric cooling systems, thereby maintaining a stable internal environment typically ranging from 2 to 8 °C. This could be further enhanced by integrating real-time temperature monitoring chips to provide continuous telemetry. At the same time, suspension-type payload fixation systems that use viscoelastic damping materials and adaptive frameworks could mitigate vibration-induced damage, thereby ensuring that medical supplies maintain their integrity throughout transport (72, 73).

### 4.2 Policy and funding level

Regulatory restrictions in many jurisdictions designate no-fly zones where emergency UAV operations require explicit authorization, yet ambiguous policy boundaries regarding emergent flight permissions often lead to cumbersome approval processes that can critically impede rescue response efficiency (74). Research on European airspace management further highlighted that incompatible national airspace data interface standards created significant interoperability gaps, where the lack of real-time data sharing consistently resultes in erroneous shortest-path calculations in cross-border trajectory optimization algorithms (75). In addition, unresolved legal and privacy concerns related to air traffic control, flight authorization, data security, and public airspace privacy, combined with regulatory gaps and inconsistent enforcement standards in China’s low-altitude UAV management, generated considerable operational uncertainty for practical implementation (76). Integrating UAVs with existing emergency response systems also presents substantial challenges, as the effective operation of AED-equipped UAVs relies on seamless coordination with dispatch centres, ground EMS personnel, and hospital emergency departments. However, mature mechanisms for information interoperability, task handover protocols, and liability delineation remain underdeveloped, which collectively impedes overall rescue efficiency.

Expanding UAV coverage through widespread deployment sites imposes prohibitive infrastructure costs, as routine maintenance, periodic flight testing, hardware refurbishment, and medical payload upkeep–including pharmaceuticals, emergency kits, AEDs, and oxygen masks–demand sustained funding. Current budgets, which often lack UAV-specific allocations and commingle resources with conventional equipment, further compromised maintenance efficacy (77). To address these challenges and continuously optimize UAV capabilities, establishing a dedicated rescue UAV fund specifically for equipment maintenance and site operations is essential. Moreover, allocating capital based on metrics such as site coverage efficiency and equipment utilization rates could enhance deployment planning, improve operational rationality, and ensure that resources are targeted effectively (78).

## 5 Limitation

This review has several limitations. Much of the current evidence is derived from simulation studies or small-scale pilot projects, which may not fully reflect the complexity of real-world emergencies. Furthermore, most published studies originate from high-income countries, limiting the generalizability of findings to resource-limited settings where UAVs may offer the greatest benefits. Future research should therefore prioritize large-scale, multi-center evaluations, incorporate diverse geographical and socioeconomic contexts, and assess long-term operational sustainability. By addressing these gaps, the field can move toward the development of resilient, equitable, and evidence-based UAV systems capable of transforming prehospital emergency care worldwide.

## 6 Conclusion

In summary, UAVs demonstrate significant potential in overcoming the spatial and temporal limitations of traditional emergency response, offering a new pathway to enhance response efficiency in remote and congested areas and reduce preventable mortality. However, existing research findings present noteworthy consistencies and contradictions. On one hand, multiple empirical studies confirm that UAVs are significantly faster than ground-based systems in delivering critical supplies such as defibrillators and hemostatic materials, with this time advantage in areas with difficult access being highly reproducible. On the other hand, their actual medical effectiveness in real-world, complex environments shows contradictions: some studies indicate that UAV intervention can significantly reduce mortality rates for specific critical conditions (e.g., cardiac arrest), while others point out that its overall cost-effectiveness is considerably influenced by operational factors such as airspace coordination and weather conditions, leading to significant variations in outcomes across different regions and scenarios. These contradictions may stem from differences in objectives between the technology validation phase and the integrated operational phase, and are also related to the current small scale of pilot projects and the lack of a standardized evaluation system.

Simultaneously, this review noted that the evidence base is heavily skewed toward high-income settings, with very few studies identified from low-income and middle-income countries (LMICs). Among these, the described challenges, including unstable infrastructure, prohibitive costs, and nascent regulatory frameworks, are not merely different but often constitute fundamental barriers to implementation that are less prevalent in high-resource contexts. These barriers directly impact the feasibility, scalability, and potential effectiveness of UAV systems in such settings. This pattern indicates that resource accessibility is likely a critical moderating factor for success, and it underscores a severe shortage of context-specific evidence needed to understand and overcome these unique obstacles in resource-limited regions.

Building on these findings, this review argues that the future development of UAV technology must adopt an inclusive global perspective and progress in a coordinated manner across three core dimensions: technology, governance, and research. Technologically, priority should be placed on enhancing all-weather operational reliability while fostering innovation in low-cost, robust, and easily maintainable solutions that are adaptable to diverse infrastructure conditions, including those prevalent in LMICs. From a governance perspective, there is an urgent need to develop adaptive airspace management frameworks and cross-sectoral coordination mechanisms that are commensurate with different national stages of development. Most crucially, the research agenda must explicitly prioritize filling the current evidence gap by supporting rigorous real-world studies and comprehensive cost-effectiveness analyses within resource-constrained environments. Such targeted research is essential to systematically evaluate operational models, overcome localized implementation barriers, and ultimately guide the equitable and effective integration of UAV technology into a heterogeneous global emergency response ecosystem.

## Data Availability

The original contributions presented in the study are included in the article/supplementary material, further inquiries can be directed to the corresponding author/s.
